# Impact of acetic acid chromoendoscopy on detection of serrated lesions in the proximal colon compared with white-light endoscopy

**DOI:** 10.1055/a-2826-9621

**Published:** 2026-04-08

**Authors:** Fernando José Savóia de Oliveira, Fernando Lander Mota, Eduardo Mendes Alves Pereira Junior, Jarbas Faraco Loureiro, Tomas Navarro-Rodriguez

**Affiliations:** 1 42522 Endoscopy, Hospital Sírio-Libanês, São Paulo, Brazil; 2 37884 Gastroenterology Department, Universidade de São Paulo Faculdade de Medicina, São Paulo, Brazil

**Keywords:** Endoscopy Lower GI Tract, Colorectal cancer, Polyps / adenomas / ..., CRC screening, Diagnosis and imaging (inc chromoendoscopy, NBI, iSCAN, FICE, CLE...)

## Abstract

**Background and study aims:**

Colorectal cancer (CRC) screening programs demonstrated a significant reduction in distal CRC mortality. However, similar results are not observed in proximal cancer, probably due to subtle and easily overlooked serrated lesions (SLs) that account for up to 30% of cases. Acetic acid chromoendoscopy may be useful in characterization of SLs. Data on its impact on SL detection rates remain limited. The aim of this study was to assess SL detection rates using high-definition white-light endoscopy (HD-WLE) versus sequential HD-WLE followed by 2% acetic acid chromoendoscopy (AAC) in the proximal colon and to evaluate the association between proximal SL rates and risk factors.

**Patients and methods:**

This prospective study included colonoscopies performed at a tertiary care center in patients aged ≥ 18 years between January and July 2021. HD-WLE was followed by HD-WLE with 2% acetic acid chromoendoscopy (AAC). SL detection rates were analyzed using McNemar's test, and risk factors were evaluated using multivariable logistic regression.

**Results:**

Four hundred thirty-four patients aged 18 to 82 years underwent both inspection strategies. Among proximal lesions, 60.9% were detected under HD-WLE and 39.1% after AAC ( *P* < 0.001). Proximal SL detection increased from 36.6% with HD-WLE to 63.4% after AAC ( *P* = 0.016). After adjustment, hypertension was associated with a higher likelihood of proximal SL detection (odds ratio 2.28; *P* = 0.032).

**Conclusions:**

HD-WLE with sequential application of 2% acetic acid chromoendoscopy was associated with higher proximal SL detection compared with HD-WLE alone. Hypertension was the only risk factor significantly associated with proximal SL detection, within the context of a sequential examination design.

## Introduction

**Fig. 1:**
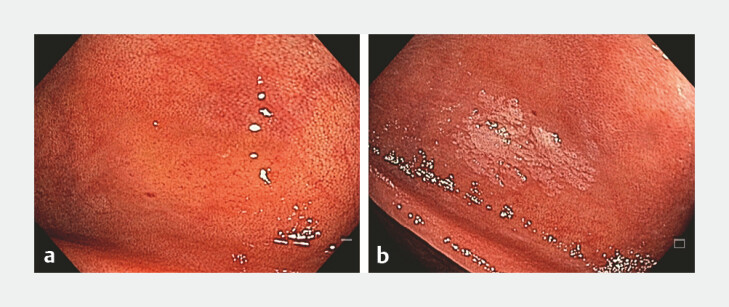
Serrated lesions often appear as subtle and easily overlooked lesions. **a** Focal loss of the submucosal vascular pattern. **b** Appearance after 2% acetic acid chromoendoscopy, allowing lesion characterization.

Colorectal cancer (CRC) is one of the most common malignancies worldwide and remains an important cause of morbidity and mortality 1 . Previously, colorectal tumors were believed to develop almost exclusively through the conventional adenoma-carcinoma sequence. However, after the serrated pathway was described, these precursor lesions are now recognized as responsible for up to 30% of CRC cases. Serrated lesions (SLs) comprise a heterogeneous group of lesions characterized by diverse morphological features, which are often subtle and easily overlooked during colonoscopy ( Fig. 1 ). Several factors seem to contribute to the wide variability in SL detection rates observed among endoscopists, including bowel preparation quality, technical aspects of the procedure, morphological features, and personal expertise 2 3 . SLs are more often located in the proximal colon and may present as sessile (0-Is), slightly elevated (0-IIa), or flat (0-IIb) lesions, according to the Paris classification 4 . Their color is similar to the adjacent mucosa, with indistinct and irregular borders often covered by a mucus layer, which frequently contributes to missed detection 5 .

Robust epidemiological evidence suggests that the challenging diagnosis of SLs may partly explain their association with synchronous and metachronous colorectal neoplasia, as well as increased risk of advanced neoplasia linked to proximally located lesions 6 7 8 9 10 11 . With growing awareness of serrated polyps, there is increasing interest in identifying potential risk factors. Reported risk factors include smoking, alcohol consumption, obesity, high-fat diet, age, sex, type II diabetes, and race 12 13 14 15 16 17 .

**Fig. 2:**
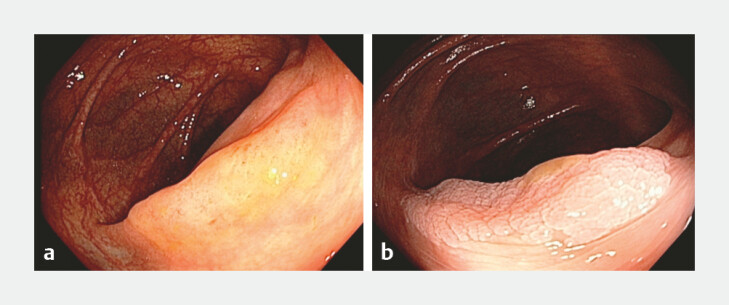
Mucolytic effect of acetic acid chromoendoscopy. **a** Slightly elevated lesion covered by mucus. **b** Appearance after 2% acetic acid chromoendoscopy.

Although high-definition white-light endoscopy (HD-WLE) remains the primary modality for diagnosing and evaluating colorectal lesions, Popoutchi et al. 18 demonstrated the usefulness of acetic acid chromoendoscopy (AAC) in the diagnosis and delineation of SLs, particularly in the right colon. Mucolytic properties of acetic acid enhance lesion visualization by removing adherent mucus and improving surface contrast ( Fig. 2 ). In addition, mild changes in pH and ionic strength induced by acetic acid interfere with protein tertiary structure, altering their optical properties and resulting in transient epithelial whitening (acetowhite reaction) 19 20 . This effect increases surface opacity and masks the subepithelial vascular network, facilitating lesion recognition.

The acetowhite reaction enhances visualization of the pit pattern of colonic lesions, thereby facilitating interpretation, and disappears after acid neutralization, indicating a temporary effect 19 . Although previous studies have combined AAC with other image-enhanced techniques for lesion characterization, evidence specifically addressing the impact of AAC on overall SL detection rates remains limited.

The aims of this prospective study were to compare detection rates for SLs in the proximal colon between HD-WLE alone and HD-WLE combined with 2% AAC. In addition, the study aimed to assess the association between proximal SL detection and clinical risk factors.

## Patients and methods

### Study design and patient selection

This was a prospective, comparative study that analyzed patients who underwent colonoscopy at a tertiary center (Hospital Sírio-Libanês, São Paulo, Brazil) between January and July 2021. Based on expert consensus, an expected detection rate of approximately 5% for proximal serrated polyps using HD-WLE was assumed for sample size calculation. Accordingly, a sample of 434 patients provided 80% power with a two-sided alpha of 0.05. Eligible participants were adults over 18 years old undergoing colonoscopy, regardless of the indication for the procedure. Exclusion criteria included refusal to participate, history of inflammatory disease, hereditary polyposis or non-polyposis syndromes, previous right colon surgery, prior diagnosis of CRC, ongoing anticoagulation therapy or antiplatelet agents, as well as allergy to acetic acid or inadequate bowel preparation 21 . All participants provided written informed consent prior to enrollment, in accordance with institutional and national ethical standards and with the Declaration of Helsinki.

### Procedure description

All patients underwent bowel preparation according to the institutional protocol, which consisted of a 10% mannitol solution combined with simethicone. Procedures were performed by experienced colonoscopists, each with a minimum of 10,000 diagnostic or therapeutic colonoscopies completed and an adenoma detection rate above 30% over the preceding year. A total of four colonoscopists participated in the study.

Colonoscopy was carried out under conscious sedation, administered by the anesthesiology team in all cases. All examinations were performed using high-definition Olympus CF-HQ190L colonoscopes coupled with the EVIS EXERA III CV-190 image processing system.

Examination of the proximal colon began with cecal intubation. The colonoscope was then withdrawn to the splenic flexure, which was defined as the anatomical landmark separating the proximal from the distal colon 22 23 24 25 26 . Under HD-WLE, any lesions or abnormalities identified during this first evaluation were treated or biopsied according to their clinical indication.

Subsequently, the colonoscope was reinserted into the cecum and an approximate total volume of 40 to 50 mL of a 2% acetic acid solution was instilled using an irrigation pump, enabling chromoendoscopy from the cecum to the splenic flexure. The proximal colon was then reexamined. All lesions identified during this second step were endoscopically resected and submitted to histopathological evaluation.

Use of antispasmodic medication was permitted on an as-needed basis at the discretion of the colonoscopist when peristaltic activity interfered with adequate inspection during withdrawal. Instillation of acetic acid did not result in clinically significant peristalsis that compromised mucosal evaluation.

### Statistical analysis and data collection

Qualitative characteristics were summarized using absolute and relative frequencies, whereas quantitative characteristics were described using summary measurements (mean, standard deviation [SD], median, minimum, and maximum). The detection rate for SLs was reported for each method and compared using McNemar’s test. SL detection rates were further evaluated according to qualitative characteristics using chi-square tests, whereas quantitative variables were compared between groups with Student’s *t* -test or the Mann-Whitney test, as appropriate.

To evaluate the association between proximal serrated lesion detection and clinical characteristics, a multivariable logistic regression model was performed, with presence of a proximal serrated lesion as the dependent variable. Independent variables included age, sex, body mass index (BMI), systemic arterial hypertension, type 2 diabetes mellitus, dyslipidemia, smoking status, and bowel preparation quality. Variables were selected based on clinical relevance and prior univariate analyses. Associations were expressed as odds ratios with corresponding 95% confidence intervals (CIs), adopting a significance level of 5%.

## Results

### Sample characterization

Of 492 patients, 434 were included in the study. Among them, 159 were undergoing their first colonoscopy and 275 had previously undergone the procedure (36.6% and 63.4%, respectively). A total of 58 patients (11.7%) were excluded for the following reasons: 21 declined participation (36.2%), 10 had a diagnosis of inflammatory bowel disease (17.2%), three had a polyposis syndrome (5.1%), seven had a prior right colectomy (12%), five had a history and/or current treatment of CRC (8.7%), and 12 were using anticoagulant agents (20.7%).

Patient age ranged from 18 to 83 years (mean 50.1 years, SD 12.5). The majority were female (269, 62%), and 86 patients (19.8%) were classified as obese according to body mass index. Most participants (364, 83.9%) self-identified as White. Regarding indication for colonoscopy, 192 (44.2%) underwent the procedure for post-polypectomy surveillance, 146 (33.6%) for CRC screening, 36 (8.3%) for abdominal pain, 26 (6.0%) for unexplained diarrhea, and 34 (7.8%) for other reasons.

With respect to comorbidities, 87 patients (20.0%) were hypertensive, 45 (10.4%) diabetic, 65 (15.0%) dyslipidemic, and 46 (10.6%) were current smokers. Bowel preparation was classified using the Boston Bowel Preparation Scale as excellent/good in 414 patients (95.4%) and fair in 20 patients (4.6%) ( Table 1 ).

**Table:** **Table 1** Baseline characteristics of patients.

Variable	Description
	(N = 434)
First colonoscopy, n (%)	
No	275 (63.4)
Yes	159 (36.6)
Age (years)	
Mean ± SD	50.1 ± 12.5
Median (min.; max.)	50 (18; 83)
Sex, n (%)	
Female	269 (62)
Male	165 (38)
Weight (kg)	
Mean ± SD	74 ± 14,7
Median (min.; max.)	72 (46; 135)
Height (m)	
Mean ± SD	1.68 ± 0,08
Median (min.; max.)	1.67 (1,5; 1,92)
BMI (kg/m²)	
Mean ± SD	26.3 ± 4,6
Median (min.; max.)	25.7 (17.3; 43)
BMI classification, n (%)	
Underweight	9 (2.1)
Normal	181 (41.7)
Overweight	158 (36.4)
Obesity class 1	66 (15.2)
Obesity class 2	16 (3.7)
Obesity class 3	4 (0.9)
Race, n (%)	
White	364 (83.9)
Multiracial	41 (9.4)
Afro-Brazilian	11 (2.5)
Asian	17 (3.9)
Indigenous	1 (0.2)
Indication for colonoscopy, n (%)	
Surveillance	192 (44.2)
Screening	146 (33.6)
Abdominal pain	36 (8.3)
Diarrhea	26 (6)
Others	34 (7.8)
Hypertension, n (%)	
No	347 (80)
Yes	87 (20)
Diabetes, n (%)	
No	389 (89.6)
Yes	45 (10.4)
Dyslipidemia, n (%)	
No	369 (85)
Yes	65 (15)
Smoking, n (%)	
No	388 (89.4)
Yes	46 (10.6)
Acetic acid allergy, n (%)	
No	434 (100)
Yes	0 (0)
Boston Scale, n (%)	
8 to 9	414 (95.4)
5 to 7	20 (4.6)
BMI, body mass index; SD, standard deviation.	

### Overall proximal lesion detection rates

A total of 340 proximal colonic lesions were identified in the study population. Of these, 207 lesions (60.9%) were detected during HD-WLE, whereas 133 lesions (39.1%) were detected only after AAC, representing a statistically significant difference favoring chromoendoscopy ( *P* < 0.001) ( Table 2 ).

**Table:** **Table 2** Total number of lesions detected by high-definition white-light endoscopy and acetic acid chromoendoscopy.

Lesion type	Method	Total	%	*P*
All proximal lesions (cecum, ascending and transverse colon)	HD-WLE	207	60.9	**< 0.001**
	HD-WLE + AAC	133	39.1	
Proximal Serrated Lesions	HD-WLE	26	36.6	**0.016**
	HD-WLE + AAC	45	63.4	
Generalized Estimating Equations (GEE) with Poisson distribution, identity link function, assuming an unstructured correlation between methods. AAC, acetic acid chromoendoscopy; HD-WLE, high-definition white-light endoscopy.				

The majority of proximal lesions detected during HD-WLE were located in the ascending colon, followed by the transverse colon and cecum.

### Serrated lesion detection rates

Regarding proximal serrated lesions, a total of 71 SLs were identified in 71 patients. HD-WLE alone detected 26 SLs (36.6%), whereas 45 additional SLs (63.4%) were detected only after AAC, demonstrating a statistically significant increase in detection (P = 0.016) ( Table 2 ).

Of the 133 additional proximal lesions identified after AAC, 45 (33.8%) were classified as SLs. These additional SLs were all < 10 mm, with most (60.0%) measuring between 1 and 5 mm. According to the Paris classification, the majority were flat or slightly elevated lesions (types 0-IIa or 0-Is) and were predominantly located in the transverse colon (53.3%), followed by the ascending colon (35.6%) and cecum (11.1%). Histopathological analysis showed that sessile SLs (SSLs) accounted for 68.9% of cases, hyperplastic lesions for 28.9%, and SSLs with dysplasia for 2.2%. No traditional SLs were detected among the additional lesions identified after AAC ( Table 3 ).

**Table:** **Table 3** Description of serrated lesions in the proximal colon detected by acetic acid chromoendoscopy.

Variable	Description
	(N = 45)
Location	
Cecum	5 (11.1)
Ascending colon	16 (35.6)
Transverse colon	24 (53.3)
Size	
1-5 mm	27 (60)
6-10 mm	18 (40)
Paris Classification	
Is	16 (35.6)
IIa	29 (64.4)
Resection	
Forceps	11 (24.4)
Cold snare	27 (60)
Hot snare	1 (2.2)
EMR	6 (13.3)
Histology	
Hyperplastic	13 (28.9)
Sessile serrated lesion	31 (68.9)
Sessile serrated lesion with dysplasia	1 (2.2)
Traditional serrated lesion	0 (0)
EMR, endoscopic mucosal resection.	

Among the SLs detected during HD-WLE, 12 (46.2%) were located in the transverse colon, 10 (38.5%) in the ascending colon, and 4 (15.4%) in the cecum. Of the SLs detected after chromoendoscopy, more than half were located in the transverse colon, whereas the ascending colon and cecum accounted for 35.6% and 11.1%, respectively.

All 45 SLs detected after AAC measured < 10 mm, with 27 (60.0%) ranging from 1 to 5 mm. SSLs accounted for 31 cases (68.9%), hyperplastic lesions for 13 (28.9%), and SSLs with dysplasia for one case (2.2%). No traditional SLs were identified. A detailed description of SL characteristics detected after chromoendoscopy is provided in Table 3 .

### SLs and associated risk factors

In unadjusted analyses, none of the evaluated clinical variables were significantly associated with proximal SL detection ( *P* > 0.05). However, in the multivariable logistic regression model, systemic arterial hypertension remained independently associated with proximal SL detection. Hypertensive patients had a 2.28-fold higher likelihood of harboring proximal SLs compared with non-hypertensive patients (95% CI 1.08–4.83; P = 0.032) ( Table 4 ).

**Table:** **Table 4** Association between detection of serrated lesions in the proximal colon and risk factors.

Variable	Proximal SLs		OR unadjusted	CI (95%)		*P*	OR adjusted	CI (95%)		*P*
	No	Yes		Inferior	Superior			Inferior	Superior	
Sex, n (%)						0.703				0.695
Female	233 (86.6)	36 (13.4)	1.00				1.00			
Male	145 (87.9)	20 (12.1)	0.89	0.50	1.60		0.88	0.48	1.64	
Age (years)			0.99	0.97	1.01	0.364	0.98	0.96	1.01	0.168
Mean ± SD	50.3 ± 12.7	48.7 ± 11.6								
Median (min.; max.)	51 (18; 83)	47.5 (26; 72)								
Race, n (%)						0.287				
White	313 (86)	51 (14)	1.00							
Multiracial	37 (90.2)	4 (9.8)	0.66	0.23	1.94					
Afro-Brazilian	11 (100)	0 (0)	-							
Asian	16 (94.1)	1 (5.9)	0.38	0.05	2.96					
Indigenous	1 (100)	0 (0)	-							
Hypertension, n (%)						0.177				**0.032**
No	306 (88.2)	41 (11.8)	1.00				1.00			
Yes	72 (82.8)	15 (17.2)	1.56	0.82	2.96		2.28	1.08	4.83	
Diabetes, n (%)						0.705				
No	338 (86.9)	51 (13.1)	1.00							
Yes	40 (88.9)	5 (11.1)	0.83	0.31	2.20					
Dyslipidemia, n (%)						0.877				
No	321 (87)	48 (13)	1.00							
Sim	57 (87.7)	8 (12.3)	0.94	0.42	2.09					
Obesity, n (%)						0.067				0.062
No	298 (85.6)	50 (14.4)	1.00				1.00			
Yes	80 (93)	6 (7)	0.45	0.19	1.08		0.43	0.17	1.04	
Smoking, n (%)						0.620				
No	339 (87.4)	49 (12.6)	1.00							
Yes	39 (84.8)	7 (15.2)	1.24	0.53	2.93					
CI, confidence interval; OR, odds ratio; SD, standard deviation; SL, serrated lesion.										

## Discussion

Our study demonstrates that HD-WLE combined with 2% AAC can increase the detection rate for SLs in the proximal colon. When applied sequentially during the same examination, AAC was associated with a significant increase in SL detection compared with HD-WLE alone. To date, this is one of the few prospective studies reporting improved proximal SL detection using this technique in a real-world clinical setting.

SLs represent a major technical challenge due to their subtle morphological features, often appearing as flat or slightly elevated lesions with pale coloration, indistinct borders, and a mucus cap, predominantly in the proximal colon 27 . These characteristics contribute to higher miss rates and may partly explain the limited effectiveness of colonoscopy in preventing proximal colorectal cancer. It is estimated that 5% to 7% of colorectal cancers are classified as interval cancers, arising from lesions missed during screening colonoscopy despite adherence to recommended surveillance intervals 28 29 30 .

Several image-enhanced endoscopic techniques have been evaluated to improve SL detection 31 . Studies comparing narrow-band imaging with HD-WLE have not consistently demonstrated superior detection rates for SLs 32 33 34 . In contrast, AAC has been shown to enhance mucosal visualization by removing adherent mucus and inducing a transient acetowhite reaction, which increases surface contrast and facilitates lesion recognition 18 19 . Tribonias et al. 35 reported higher detection rates when acetic acid was used during a second inspection compared with saline, supporting a potential additive effect beyond reinspection alone.

Nevertheless, the sequential design of our study does not allow complete exclusion of a tandem observation effect. The act of reexamining the same colonic segment is known to increase lesion detection, particularly for conventional adenomas. However, prior evidence suggests that tandem inspection has a more limited impact on SLs compared with adenomas. The magnitude and lesion-specific nature of the increase observed in our study, restricted to proximal SLs, supports a biologically plausible additive role of acetic acid, rather than a nonspecific benefit of a second inspection alone.

Regarding clinical risk factors 36 37 , hypertension emerged as the only variable independently associated with proximal SL detection after multivariable adjustment. Although the CIs were relatively wide, reflecting a limited number of events, this finding is consistent with emerging evidence linking metabolic risk factors to serrated neoplasia 36 . These results should be interpreted cautiously and warrant confirmation in larger, adequately powered studies.

Some practical limitations and disadvantages of AAC should be acknowledged. Transient colonic spasm and increased mucus production have been described with acetic acid use. In our experience, these effects were infrequent and did not compromise mucosal evaluation. When necessary, additional suction, irrigation, or on-demand antispasmodic agents were sufficient to maintain adequate visualization.

This study has additional limitations. Colonoscopists were not blinded to acetic acid use, and the study was not randomized. Each patient served as their own control, which may introduce operator-related bias. Nonetheless, this design reflects routine clinical practice and allows assessment of the incremental value of acetic acid when applied during a systematic reinspection of the proximal colon.

## Conclusions

In summary, this prospective study suggests that sequential use of 2% AAC during HD-WLE is associated with improved detection of proximal SLs. Although acknowledging the influence of reinspection, simplicity, low cost, safety profile, and ease of application of acetic acid supports its potential role as an adjunctive tool in daily colonoscopic practice, further randomized controlled trials with alternating inspection sequences are needed to isolate its independent effect on SSL detection.
